# Classifying Step and Spin Turns Using Wireless Gyroscopes and Implications for Fall Risk Assessments

**DOI:** 10.3390/s150510676

**Published:** 2015-05-06

**Authors:** Peter C. Fino, Christopher W. Frames, Thurmon E. Lockhart

**Affiliations:** 1 Department of Mechanical Engineering, Virginia Polytechnic Institute and State University, Blacksburg, VA 24061, USA; E-Mail: fino@vt.edu; 2 Department of Industrial and Systems Engineering, Virginia Polytechnic Institute and State University, Blacksburg, VA 24061, USA; E-Mail: cframes5@vt.edu; 3 School of Biological and Health Systems Engineering, Ira A. Fulton Schools of Engineering, Arizona State University, Tempe, AZ 85287, USA

**Keywords:** gait, turning, wireless sensors, IMU, fall risk

## Abstract

Recent studies have reported a greater prevalence of spin turns, which are more unstable than step turns, in older adults compared to young adults in laboratory settings. Currently, turning strategies can only be identified through visual observation, either in-person or through video. This paper presents two unique methods and their combination to remotely monitor turning behavior using three uniaxial gyroscopes. Five young adults performed 90° turns at slow, normal, and fast walking speeds around a variety of obstacles while instrumented with three IMUs (attached on the trunk, left and right shank). Raw data from 360 trials were analyzed. Compared to visual classification, the two IMU methods’ sensitivity/specificity to detecting spin turns were 76.1%/76.7% and 76.1%/84.4%, respectively. When the two methods were combined, the IMU had an overall 86.8% sensitivity and 92.2% specificity, with 89.4%/100% sensitivity/specificity at slow speeds. This combined method can be implemented into wireless fall prevention systems and used to identify increased use of spin turns. This method allows for longitudinal monitoring of turning strategies and allows researchers to test for potential associations between the frequency of spin turns and clinically relevant outcomes (e.g., falls) in non-laboratory settings.

## 1. Introduction

Falls in elderly populations constitute a significant economic and healthcare burden, accounting for $7.8 billion USD in 2002 [1] and between $1,000 to $10,000 USD per fall [2]. Falls resulting from slips are more common during turning than straight walking [3] and falls while turning are 7.9 times more likely to result in hip fracture than falls during straight walking [4]. Additionally, turning and non-straight steps can account for up to 45% of daily locomotion [5]. Despite the high prevalence of turning and increased risk of falls and injuries, most fall assessment and prevention studies have not considered turning as a potential indicator for fall risk or as a predictor of fall outcomes.

Turning can be classified into two disparate strategies: spin turns (ipsilateral turns) and step turns (contralateral turns) [6,7,8]. Spin turns are characterized by turning on the ipsilateral limb (e.g., left turn while the left limb is the stance limb) and step turns are characterized by turning on the contralateral limb (e.g., left turn while the right limb is the stance limb). Of these two strategies, spin turns are considered less stable than step turns because the whole-body center-of-mass (COM) remains outside the base-of-support (BOS) for the majority of the stance phase [9,10]. Because of this COM displacement, one could expect step turns to be more prevalent.

While young adults prefer step turns [11], this preference declines in older populations. Older adults use a higher proportion of spin turns compared to younger adults [6,12]. Additionally, fall-prone elderly have a higher frequency of spin turns in a multi-target stepping task compared to healthy elderly [13]. Because of the increased frequency of spin turns with age, the prevalence of spin turns in everyday activities may therefore be a predictor of fall risk and could be a useful metric for fall risk assessments. However, visual classification (*i.e.*, a researched viewing the turn in person or on video to classify the turn) is the only existing method to identify step and spin turns, limiting turning strategy research to laboratory or video recorded settings.

As non-invasive wireless inertial measurement units (IMUs) are being increasingly used to monitor gait characteristics to determine the fall risk of elderly individuals [14,15,16], the same sensors could be used to automatically differentiate step and spin turns remotely, providing methods for turning research outside a laboratory and a potentially useful element for fall monitoring and prevention systems. This article presents a method to remotely classify turning strategy using raw gyroscopic data from IMUs for use in further turning research and fall monitoring applications. A preliminary version of this data was previously presented [17].

## 2. Experimental Section

### 2.1. Participants

Five healthy young adults (four male, one female) 22–28 years of age (mean age 24.6 ± 2.4 years, mean height 1.79 ± 0.12 m, mean weight 77.3 ± 10.0 kg) were recruited from Virginia Tech and the surrounding community for the study. Participants were informed of the protocol and signed an informed consent form prior to the experiment. Participants were excluded if they had any history of balance disorders, dizziness, musculoskeletal injury the past year affecting normal gait, any neurological disorders, one or more concussions within the past year, and/or significant visual impairment. The complete protocol was approved by the Institutional Review Board at Virginia Tech.

### 2.2. Instrumentation

**Figure 1:**
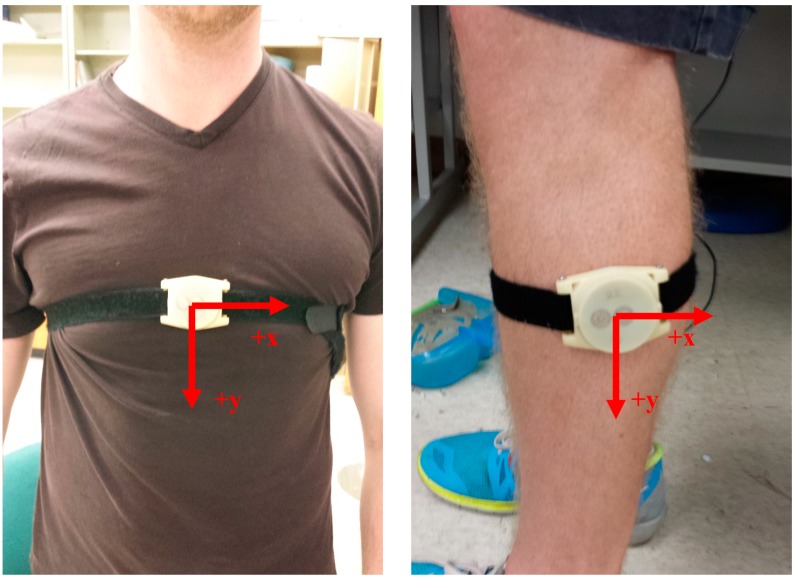
Example placement of the IMU’s: over the sternum and laterally on the mid-shank. Each IMU was oriented using a left-hand coordinate system.

Data were collected using three wireless IMUs each consisting of a MMA7261QT tri-axial accelerometer, an IDG-300 (*x* and *y* plane gyroscope) and an ADXRS300 *z*-plane uniaxial gyroscope. All components were aggregated in the Technology Enabled Medical Precision Observation (TEMPO) platform which was manufactured in collaboration with a research team at the University of Virginia [18,19]. The IMU nodes communicated through a Bluetooth adapter to a desktop computer through a custom built program in LabView (National Instruments, Austin, TX, USA). The IMUs were strapped on the participants’ trunk at the sternum level and on both shanks as shown in Figure 1. The IMUs were aligned with the *y-*axis facing downward and followed a left-handed coordinate system. Data were sampled at 128 Hz.

### 2.3. Experimental Procedure

The full procedure was reported by Fino and Lockhart [20]. Briefly, participants performed a series of 90° turns following a 0.75 m wide marked path at three self-selected walking speeds: normal (NW), slower than their normal pace (SW), and “as fast as possible without running or jogging” (FW). The corner was marked with a 10 cm diameter pylon of various height (0 cm, 63 cm, 104 cm, and 167 cm).

Participants walked within the path and around the corner until they reached a stop line located 2.5 m after the turn. Participants performed 24 turning trials for each speed. The turning trials were divided into four blocks, one for each obstacle height. For each obstacle height, participants performed three step turns and three spin turns by changing the starting foot for each trial. To eliminate order effects, speed, obstacle height, and step turn *versus* spin turn order was rotated for each participant [20]. A total of 72 turning trials were recorded for each participant: three spin turns and three step turns for each of the four obstacle heights at each of the three speeds.

### 2.4. Data Analysis

**Figure 2:**
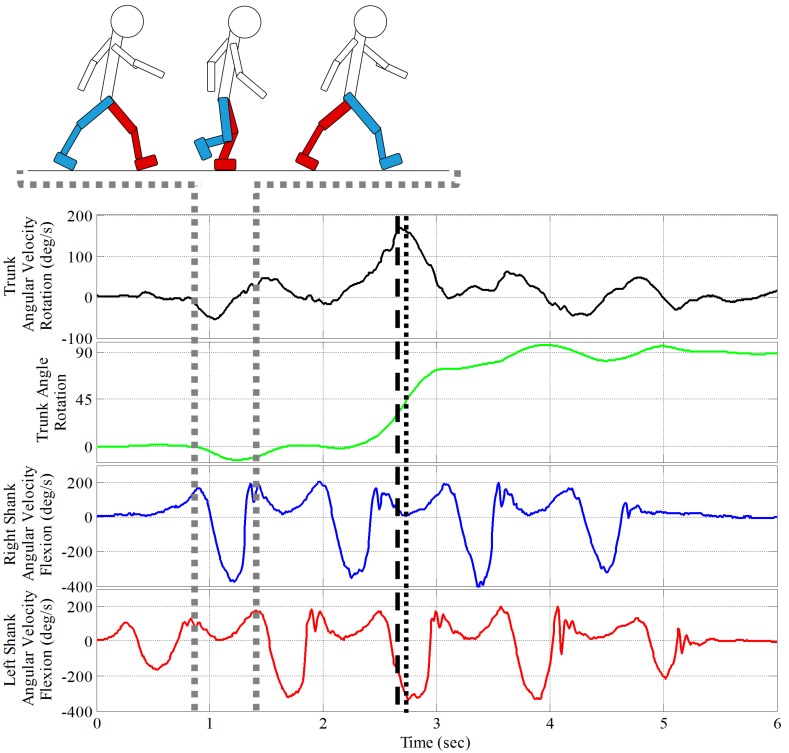
Trunk (top, black), right shank (middle, blue), and left shank (bottom, red) angular velocities during a step turn to the left. The trunk angle obtained by integrating the trunk angular velocity is shown in green. The black dashed line indicates the peak trunk angular velocity (PM). The black dotted line indicated the time when the participant turned 45° (IM). Both times occur during the stance phase for the right foot and the swing phase for the left foot, indicating a step turn. The small oscillations before and after the peak trunk angular velocity represent steps before and after the turn.

All 72 trials from all five participants were analyzed. All analyses were performed using MATLAB (MATLAB and Statistics Toolbox Release 2014a, The MathWorks, Inc., Natick, MA, USA). Two separate classification methods were used to analyze the raw, unfiltered IMU data, shown in Figure 2. The first method, hereby referred to as the peak method (PM), used the magnitude of the shanks’ angular velocity at the time of the peak trunk rotational velocity to classify step and spin turns. The trunk rotated axially with each step, resulting in small oscillations in the angular velocity, but a change in direction (*i.e.*, turn) produced a noticeably larger angular velocity as the trunk reoriented. For each trial, the time of the peak trunk rotational velocity in the transverse plane (*y*-axis), indicating a turn, was detected. The shanks’ rotational velocities in the sagittal plane (*z*-axis) were then compared at that instant in time to differentiate the stance limb and swing limb. The shank, left or right, with the greater magnitude of rotational velocity was classified as the swing limb. The shank with the lower magnitude rotational velocity at that time was classified as the stance limb. The direction of the turn was compared to the stance limb to determine the turning strategy.

The second classification method, referred to as the integrated method (IM), used the integrated gyroscope signal to identify the time of the turn. The rotational velocity was integrated using trapezoidal integration to give the heading angle relative to the initial position. When this angle exceeded half the angle of the turn (*i.e.*, 45° for a 90° turn), the shanks’ rotational velocities were compared and the turning strategy was determined using the same procedure as above. Each IMU classification method was then compared to the visual classification performed by the researcher at the time of the data was collected for each trial.

The two different IMU methods were then combined to create a third method, the combined method (CM) by discarding trials with disagreement between the PM and IM. If the result for the PM and IM method agreed (e.g., both output step turns), then that trial was retained in the CM. Trials with disagreement between PM and IM were excluded from the CM.

## 3. Results

The PM classification results are shown Table 1. Table 2 shows the classification results the IM, and Table 3 shows the results of the CM. When the two methods were combined, the methods disagreed on 102 total turns (51 step turns, 51 spin turns), which were excluded from the CM analysis. Overall, the PM correctly classified 76.4% of the trials. The IM was slightly more accurate, correctly classifying 80.3%, and the CM classified 89.5% of the trials correctly when the PM and IM agreed.

**Table 1:** Classification results using the PM method. The number of trials correctly classified by the IMU are separated by strategy and shown. Sensitivity and specificity were calculated with respect to spin turns. The overall accuracy was calculated as the percentage of overall of trials correctly classified.

	Visual Classification		Correct IMU Classification		Sensitivity	Specificity	Overall Accuracy
	Spin	Step	Spin	Step	(to Spin Turns)		
**Overall**	180	180	137	138	76.1%	76.7%	76.4%
**Slow**	60	60	47	51	78.3%	85.0%	81.7%
**Normal**	60	60	48	45	80.0%	75.0%	77.5%
**Fast**	60	60	48	36	80.0%	60.0%	70.0%
**0 cm**	45	45	37	33	82.2%	73.3%	77.8%
**63 cm**	45	45	35	33	77.8%	73.3%	75.6%
**104 cm**	45	45	39	32	86.7%	71.1%	78.9%
**167 cm**	45	45	34	32	75.6%	71.1%	73.3%

**Table 2:** Classification results using the IM method. The number of trials correctly classified by the IMU are separated by strategy and shown. Sensitivity and specificity were calculated with respect to spin turns. The overall accuracy was calculated as the percentage of overall of trials correctly classified.

	Visual Classification		Correct IMU Classification		Sensitivity	Specificity	Overall Accuracy
	Spin	Step	Spin	Step	(to Spin Turns)		
**Overall**	180	180	137	152	76.1%	84.4%	80.3%
**Slow**	60	60	49	53	81.7%	88.3%	85.0%
**Normal**	60	60	44	45	73.3%	75.0%	74.2%
**Fast**	60	60	44	54	73.3%	90.0%	81.7%
**0 cm**	45	45	35	40	77.8%	88.9%	83.3%
**63 cm**	45	45	36	36	80.0%	80.0%	80.0%
**104 cm**	45	45	34	37	75.6%	82.2%	78.9%
**167 cm**	45	45	36	36	80.0%	80.0%	80.0%

**Table 3:** Classification results using the CM method. The number of trials correctly classified by the IMU are separated by strategy and shown. Sensitivity and specificity were calculated with respect to spin turns. The overall accuracy was calculated as the percentage of overall of trials correctly classified.

	Visual Classification *		Correct IMU Classification		Sensitivity	Specificity	Overall Accuracy
	Spin	Step	Spin	Step	(to Spin Turns)		
**Overall**	129	129	112	119	86.8%	92.2%	89.5%
**Slow**	47	43	42	43	89.4%	100.0%	94.4%
**Normal**	41	49	32	44	78.0%	89.8%	84.4%
**Fast**	41	37	38	32	92.7%	86.5%	89.7%
**0 cm**	30	31	29	29	96.7%	93.5%	95.1%
**63 cm**	30	32	24	32	80.0%	100.0%	90.3%
**104 cm**	35	31	32	27	91.4%	87.1%	89.4%
**167 cm**	34	35	27	31	79.4%	88.6%	84.1%

* Only trials with agreement between the PM and IM classification methods were compared.

## 4. Discussion

Using three wireless IMU’s, the automatic classification of step and spin turns was relatively accurate when compared to the visual classification of the researchers. When combining the methods, the classification approached 90% accuracy. Individually, both the PM and IM struggled to identify the correct stance limb when the designated time point (peak or integrated angular velocity exceeding 45°) occurred near heel-contact or toe-off of either foot. At these times in the gait cycle, both feet are in contact with the ground (double-stance) and the angular velocities of both shanks are similar and small. Therefore, the PM and IM methods, which rely on the instantaneous angular velocity of the shank at a single point in time, will have difficulty identifying a definitive stance limb. However, in the CM, two points in time are considered, the times for both PM and IM. Using two separate, nearby, epochs, the CM reduces the influence of spurious rotational velocity fluctuations at heel contact and toe-off. By y considering only the trials with agreement between the two methods, the CM removed many of the trials in question and resulted in a more accurate classification scheme, albeit with a reduced sample.

The CM performed the best when the participants walked slowly (0.91 m/s), correctly identifying 89.4% of spin turns and 100% of step turns. The average slow walking speed here closely matches the average gait speed of community-dwelling older adults, which ranges from 0.4 m/s to 1.4 m/s, with an average velocity of 0.92 m/s [21]. Even though young adults were used in this study to demonstrate the classification methods, the CM’s excellent performance at slow gait speeds suggests this method is applicable for research in community-dwelling elderly populations, such as fall risk assessments.

Current attempts to quantify fall risk using IMU’s mostly rely on quantifying gait parameters or standard tasks (e.g., sit-to-stand and timed up and go) [15,16] but do not consider turning. Since slips and falls are more common during turning than straight walking [3] and a higher incidence of hip fracture occurs in falls while turning [4], it seems natural to consider turning in a fall risk assessment.

This simple method of classifying the turning strategy (step *versus* spin) using wireless IMU’s may provide a useful metric in the overall assessment of an individual’s fall risk: the frequency of step turns and spin turns in everyday locomotion. In a laboratory setting, elderly individuals utilized a spin turn between 40% and 45% of the time when walking at their normal speed, with up to a 61% frequency of spin turns while walking slower than normal [6]. Additionally, Yamada *et al.* [13] reported a greater frequency of spin turns during a multi-target stepping task in elderly with a high risk of falling compared to healthy elderly. The higher frequencies of spin turns are concerning because the COM is laterally displaced outside the BOS more during spin turns than step turns [9,10] indicating a high risk of lateral falls if a perturbation (slip, trip) occurs.

The choice of a spin turn over a more stable step turn in elderly is not fully understood, but may be influenced by the physiological demands of each turn. Courtine *et al.* [22] reported increased stance limb muscle activation amplitudes in the soleus, gastrocnemius medialis, tibialis anterior, and gluteus medialis for the outer limb (step turn) during curved walking compared to the inner limb (spin turn). However, the inner limb had higher muscle activation amplitudes in the biceps femoris and gastrocnemius lateralis compared to the outer limb. It is possible that a prevalence of spin turns may be indicative of a change in musculature (e.g., a decline in soleus strength). However, this potential association has never been tested in part because of the challenges of longitudinally monitoring turning strategies.

The classification method presented here can be used without direct visual observation to investigate whether such associations exist between spin turns and clinically relevant outcomes (e.g., falls, declining strength) in non-laboratory settings. Following such studies, this metric may prove useful in identifying subtle gait changes or limb asymmetries which may coincide with individuals changing their preference from one strategy to another. Additionally, this simple monitoring can prove useful in clinical rehabilitation settings following lower limb injury or surgery. Overall, the turning classification method presented here is valuable to researchers wishing to examine turning where visual observation, either in person or through video, is limited, cumbersome, or unavailable, such as participants’ homes, outdoors, *etc*.

Notably, the methods presented here used only one axis of each gyroscope to minimize the size and cost of this method for future uses. Incorporating several other signals may further increase the accuracy of the system. However, by only using uniaxial gyroscopes, the overall cost, size, and battery consumption of each sensor can be minimized. Gyroscopic drift presents a slight limitation when using these methods for longitudinal observation, but that limitation can be overcome by only comparing a small sliding window of strides (*i.e.*, the most recent 6 strides) to limit the overall drift within the window of calculation. Additionally, more advanced classification algorithms that examine more detailed time-histories should be attempted to further increase the accuracy, especially during multi-step turns.

## 5. Conclusions/Outlook

In conclusion, a simple algorithm to accurately differentiate step and spin turns was presented using three wireless IMUs. Though the CM was not 100% accurate, its simplicity and the remote non-invasive sensors prompt rapid implementation into wireless fall prevention systems and other gait monitoring programs. The importance of step or spin turns in an individual’s gait has received relatively little attention. Implementation and utilization of this system or similar methodologies can lead to increased knowledge about preferred turning strategies. Additionally, this method allows researchers to longitudinally investigate potential associations between turning strategies and clinically relevant outcomes.
